# The Stem Cell Niche: Interactions between Stem Cells and Their Environment

**DOI:** 10.1155/2018/4879379

**Published:** 2018-10-14

**Authors:** Sari Pennings, Karen J. Liu, Hong Qian

**Affiliations:** ^1^Centre for Cardiovascular Science, Queen's Medical Research Institute, University of Edinburgh, Edinburgh EH16 4TJ, UK; ^2^Centre for Craniofacial and Regenerative Biology, King's College London, London SE1 9RT, UK; ^3^Center for Hematology and Regenerative Medicine, Department of Medicine, Karolinska Institute, Karolinska University Hospital, Stockholm, Sweden

Stem cells constitute a source of self-renewing cells with a potential to differentiate into distinct tissues. In the embryo, these cells supply the multiple different cell lineages necessary to generate functional organs. Adult tissues retain somatic stem cells with capabilities for specific tissue turnover and repair. Embryonic and adult stem cell research has shown that stem cell fates are controlled by their specialized microenvironment, referred to as the stem cell niche, via direct cell-cell interactions and the molecular signals emitting from the niche. The niche is formed by the ensemble of stromal cells and the factors they produce, including adhesive signals, soluble factors, and matrix proteins (Figure 1). While we have some understanding of the interactions between adult stem cells and their environment, the requisite components of the stem cell niche are still unclear. Furthermore, tissue-specific stem cells are likely to reside in specialized niches that require further characterisation in each tissue. Progress towards understanding and building a stem cell niche will be necessary to advance *in situ* applications of *in vitro* reprogrammed pluripotent stem cells, differentiated stem cells, and targeted tissue-specific stem cell expansion in tissue regeneration. This may additionally lead to better understanding of how abnormal microenvironments, such as the leukaemic stem cell niche, can contribute to cancer initiation and progression.

Schofield first postulated the hypothesis of a specialized stem cell niche for haematopoietic cells [1]. Since then, a range of stem cell niches regulating tissue turnover and maintenance has been identified and characterised. Even adult tissues previously regarded as postmitotic are now known to be maintained by low levels of steady-state cell replacement during the life course; however, this may not be sufficient under pathological conditions of injury or degenerative diseases. Research on the mechanisms underlying stem cell niche regulation and the strategies to replicate such natural microenvironments *in vitro* could be used to expand stem cells *ex vivo* without losing their native properties.

The stem cell niche typically has a spatial organisation that provides anatomical and functional interactions contributing to stem cell fate specification as well as maintenance of existing clones. These interactions are mutual and dynamic. Stem cells, particularly transformed cancer stem cells, can determine or reprogramme their niche. Stem cell plasticity in response to injury is contained within this environment instructive of stem cell fates [2]. On the other hand, many stem cells show a decline in function over the lifetime, which may underlie the ageing process in organisms [3]. The contribution of the microenvironment to stem cell fate bias is still unclear and requires further investigation. Considerable challenges remain in deducing commonalities, as well as stem cell niche-specific mechanisms, amongst the variety of stem cell supportive microenvironments.

As it is becoming clearer that the niche contributes to the maintenance of stem cell identity, the study of both is needed for understanding and recreating stem cell properties. What links these various stem cell niches? During the development of an organism, morphogenetic cell movements and proliferation lead to the formation of the organs in the body. For the main populations of progenitor cells, this process involves a gradual specification induced by the developmental signals they encounter in their changing cellular environments, as they divide and expand into tissues. After completion of mammalian development, some multipotent progenitors and stem cells remain dedicated to tissue turnover in organs. Nested in various tissue-specific locations, they can undergo a range of cell fate changes essential for tissue homeostasis. Niches are typically remnants not of the early organ fields but of later stem cell locations in organogenesis, e.g., bone marrow, sutures in a bone [4, 5].

Cancer stem cells can determine their own *de novo* niche formation during cancer progression, showing that the niche mechanisms are at risk of misappropriation and alterations [6, 7]. The well-studied niches for haematopoietic stem cells, intestinal stem cells, and skin stem cells, as well as the examples of the hair follicle, mammary gland, and neural stem cell niches, have shown that tissue embedded adult stem cell states can include actively dividing cells as well as cells in a state of quiescence [8]. Such states are controlled by signalling in the niche. Embryonic stem cells can also adopt different stem cell states, depending on culture conditions mimicking the signalling conditions of embryonic environments at either blastocyst or epiblast stages; yet, cultured cells show epigenetic changes compared to their embryonic counterparts [9]. The responsiveness of adult stem cells and embryonic stem cells to their environment offers the prospect of bioengineered niches recapitulating developmental potential for biomedical applications [10].

This special issue presents novel research and concepts that link *in vivo* stem cell function to the niche, including research on *in vitro* stem cell niche modelling. An introduction to the adult stem cell niche is provided by S. Bardelli and M. Moccetti, who review recent advances in translational medicine approaches aiming to mimic the natural adult stem cell niche for regenerative medicine. Advances in intestinal stem cell (ISC) niche research are reviewed by L. Meran et al. from the Li group, focusing on the extracellular and cellular niche components; N. Gjorevski and P. Ordóñez-Morán summarizing recent studies on *in vivo* and *in vitro* models of the ISC niche; and B. C. E. Peck et al. from the Sethupathy group presenting the mutual interactions between the ISC niche and gut microbiota and reviewing the available tools to study these interactions. There is increasing recognition of the importance of the bone marrow vascular niche in stem cell regulation in the bone. S. K. Ramasamy reviews recent advances in understanding the heterogeneity and structure of the blood vessels in the bone and their functions in regulating mesenchymal and haematopoietic stem cells. A. Mauretti et al. from the Bouten group and C. Aguilar-Sanchez et al. from the Pennings group provide updates on the debate regarding the function, occurrence, and microenvironment of cardiac progenitor cells.

The cancer stem cell niche may contribute to cancer progression and resistance against chemotherapy, presumably through niche protection of cancer stem cells that are considered a root cause of the cancer relapse. Strategies targeting interactions between cancer stem cells and their niche are discussed with respect to pancreatic cancer by J. Zhao et al. and ovarian cancer by M. Varas-Gody et al. Mesenchymal stem cell (MSC) research reports by D. Aboalola and A. Youssef in the Han laboratory show their regulation by insulin growth factor and its binding protein, as well as at the onset of osteogenic differentiation and myogenic differentiation, respectively. The role of MSC microRNAs in extracellular vesicles promoting skin repair is investigated by A. da Fonseca Ferreira et al. in the Assis Gomes laboratory. A circadian dynamics study by E. H. Rogers et al. in the Hunt lab shows interesting differences between MSCs originating from different tissues, which may be relevant to tissue engineering and stem cell therapies. Isolation techniques for corneal stroma-derived cells are functionally compared by R. Nagymihály et al. from the Petrovsky laboratory indicating changes in the expression profile of markers compared to the *in situ* state of these stem cells. H. Albalushi et al. from the Stukenborg laboratory report on the stabilising effects on human ES cells when grown on new laminin 521 substrates providing a more controllable culture microenvironment.

## Figures and Tables

**Figure 1:**
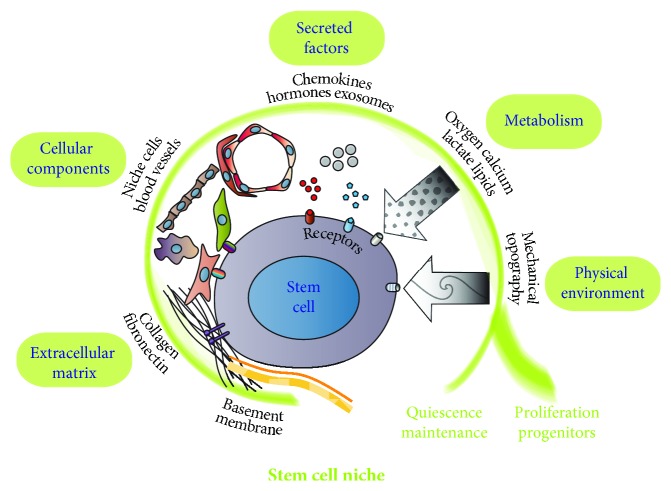
Schematic diagram of the stem cell niche. Shown here is a stem cell with the interacting factors and components of the microenvironment known to regulate resident stem cells, which can be maintained as proliferating or quiescent cells. The actual niche architecture and components may vary for different types of embryonic, adult stem cells and progenitor cells. Not drawn to scale are examples of various cellular niche elements (fibroblasts, MSCs, pericytes, endothelial cells, macrophages, tissue-specific niche architecture cells, and the interacting receptors); extracellular matrix (collagen, fibronectin, basement membrane, and the interacting integrins); secreted factors (exosomes, chemokines, hormones, and the signalling receptors); metabolism producing nutrients and redox state and their channels and transporters; and physical forces within the niche architecture and the corresponding mechanoreceptors.
